# Elucidation of Softening Mechanism in Rinse-Cycle Fabric Softeners. Part 2: Uneven Adsorption—The Key Phenomenon to the Effect of Fabric Softeners

**DOI:** 10.1007/s11743-016-1815-x

**Published:** 2016-04-19

**Authors:** Takako Igarashi, Koichi Nakamura, Masato Hoshi, Teruyuki Hara, Hironori Kojima, Masatsugu Itou, Reiko Ikeda, Yoshimasa Okamoto

**Affiliations:** R&D-Household Products Research, Kao Corporation, 1334 Minato Wakayama-shi, Wakayama, 640-8580 Japan; R&D-Analytical Science Research, Kao Corporation, 1334 Minato Wakayama-shi, Wakayama, 640-8580 Japan

**Keywords:** Fabric softener, Mechanism, Cotton, Bound water, Hydrogen bonding, Cross-linking, Gradation, Bromophenol-blue coloration method

## Abstract

We investigated the actual factor determining the softening effect of a fabric softener. The adsorption area of the softener on model cotton cloths and yarns was identified using bromophenol blue. There was almost no softener at the cross-points of the yarns in the cloth samples or in the inner part of the yarns. The softening performance was better when there was less softener at the cross-points of the yarns than when the yarns were evenly covered by the softener. Thus we conclude that the presence of softener at the cross-points of yarns is not a vital factor in the softening effect. In addition, more softener was found on the outer part of the yarn than the inner part, indicating gradation in the adsorption pattern of the softener. Thus, we propose that more softener is adsorbed on the exposed part of the yarn in a cloth, and the formation of a hydrogen-bonding network containing bound water is inhibited, thus softening the outer part of the yarn. However, the presence of a small amount of softener in the inner part of the yarn preserves the hydrogen-bonding network. Favorable elasticity, or bounce, of the yarns and cloth is realized when an appropriate amount of softener is used. Excess softener would reach the inner part of the yarn, reducing the diameter of the core part of the yarn, making the cloth appear wilted.

## Introduction

**Fig. 1:**
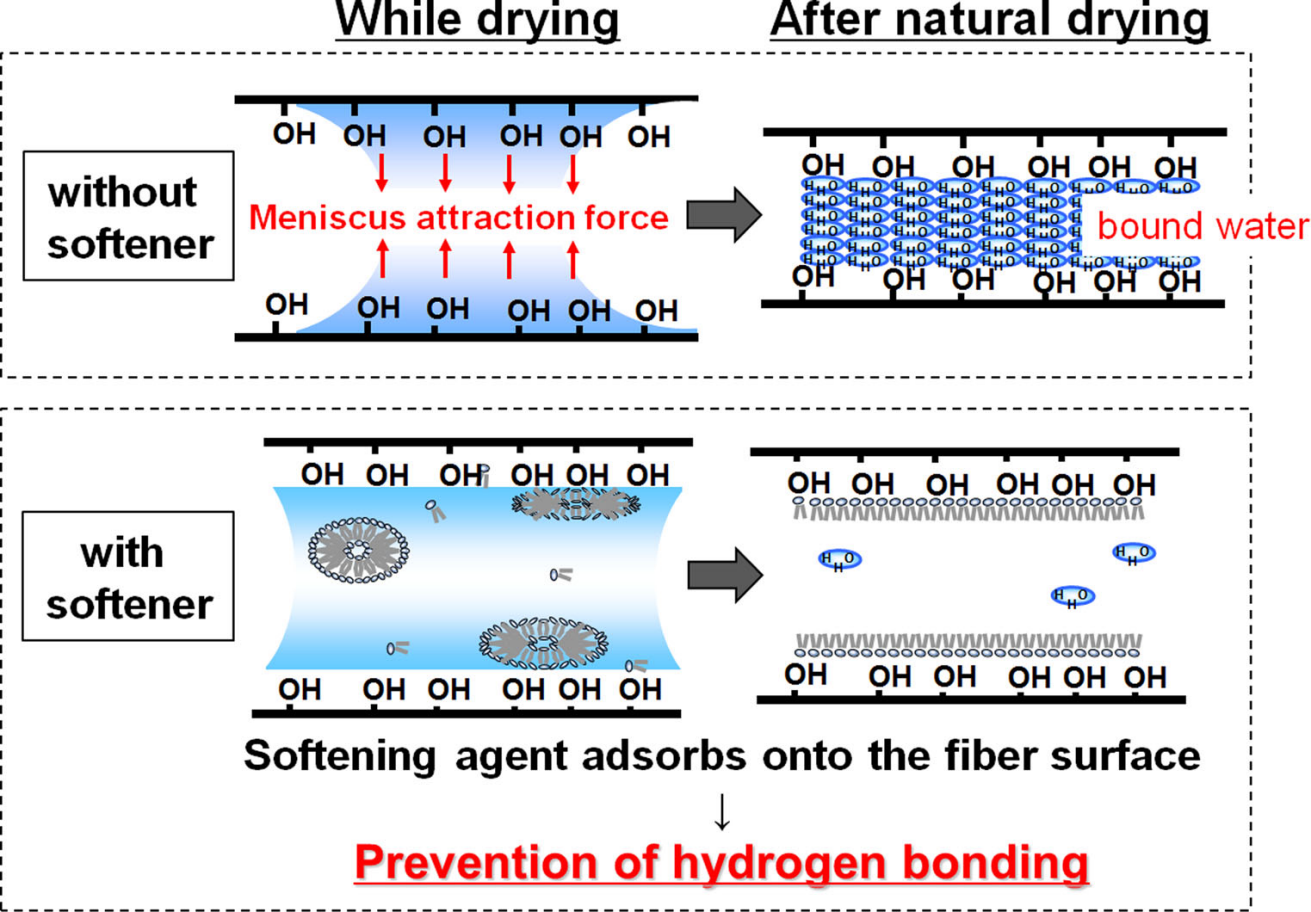
Image of softening mechanism in the use of a softener

Enhanced lubrication between the fibers of a cloth or a fabric has been proposed as the mechanism underlying the softening effect of a fabric softener. Here we offer a new theory for the softening mechanism on the basis of two main phenomena in our study using cotton fibers and cloth samples: (1) excessive hardness of cotton cloths and yarns that were subjected to natural drying; (2) apparent softening of untreated yarns after complete drying [1, 2], as though they had been treated by a fabric softener. Based on these results, we propose that: (1) the excessive hardness of fabrics after natural drying is due to cross-linkage between the cotton fibers as a result of hydrogen bonding caused by bound water; (2) the inhibition of this cross-linkage by the softener results in the softening effect (Fig. 1).

There has been much discussion on the mechanism of adsorption of softeners on cotton fibers. The most widely accepted explanation is that the cationically charged vesicles of the softener are adsorbed on the surface of the anionically charged cotton fibers [3, 4]. However, there is uncertainty associated with this mechanism, because the sequence of adsorption is thermodynamically unfavorable: adsorption is followed by the formation of multilayers between the fibers and water by the cationic surfactants, which are sparingly soluble in water, together with subsequent formation of a monolayer between the fibers and air. Crutzen [5] suggested that hydrophobic interactions derived from the long alkyl chains of cationic surfactants are an important driving force in adsorption, because the softener can be adsorbed even on uncharged fiber surfaces. Minegishi [6] reported that the time required to attain adsorption equilibrium is strongly influenced by a mechanical force when the softener is adsorbed on cotton fibers. This finding also indicates that the dominant factor is not electrostatic interactions, but the “collision” between the fibers and vesicles of the softener. Okumura [7] reported that fabric softener adsorbed on the surface of cotton maintains uniform multilayered states. Nakamura et al. [8] reported that the softener exists as an “interdigitated structure” after the collapse of vesicles during drying. Sakai et al. [9] recently used atomic force microscopy to observe the adsorption on the surface of mica, and reported that the vesicles adsorbed by collision immediately expanded to form a double-layered molecular membrane. The adsorption state of the softener molecules was then maintained without loss of the molecules from the surface, because of the electrostatic interaction between the anionic part of the cotton surface and the cationic moiety of the softener molecule.

In this study, we aimed to deepen our understanding of the adsorption behavior of a fabric softener by using a cationic surfactant indicator to focus on the assembled cotton fibers (yarn and cloth). We also considered the reason for the softening effect based on our findings, some of which were reported in our previous papers [1, 2], while others are presented here for the first time. The results show that the existence of the softener at the cross-points of the yarns in the cloth has no direct role in the “softness” of the cloth, leading us to conclude that reduced friction or lubrication at the cross-points is not the main reason for the softness caused by fabric softeners.

## Experimental Section

### Samples

#### Thick Cotton Yarn

Cotton knitting yarns (diameter: 2.7 mm; Amiami Cotton, manufactured by Hamanaka Corp., Japan) were used as samples for the adsorption experiments. Before the experiments, 18 g of the yarns was thoroughly pre-washed with 1000 mL of a solvent [CHCl_3_/MeOH = 1/1 (wt. ratio)] and stirred for 5 min. This pre-washing process was carried out three times to remove the pre-treatment oils adsorbed on the yarn during the yarn-making process.

#### Model Cotton Cloths

**Fig. 2:**
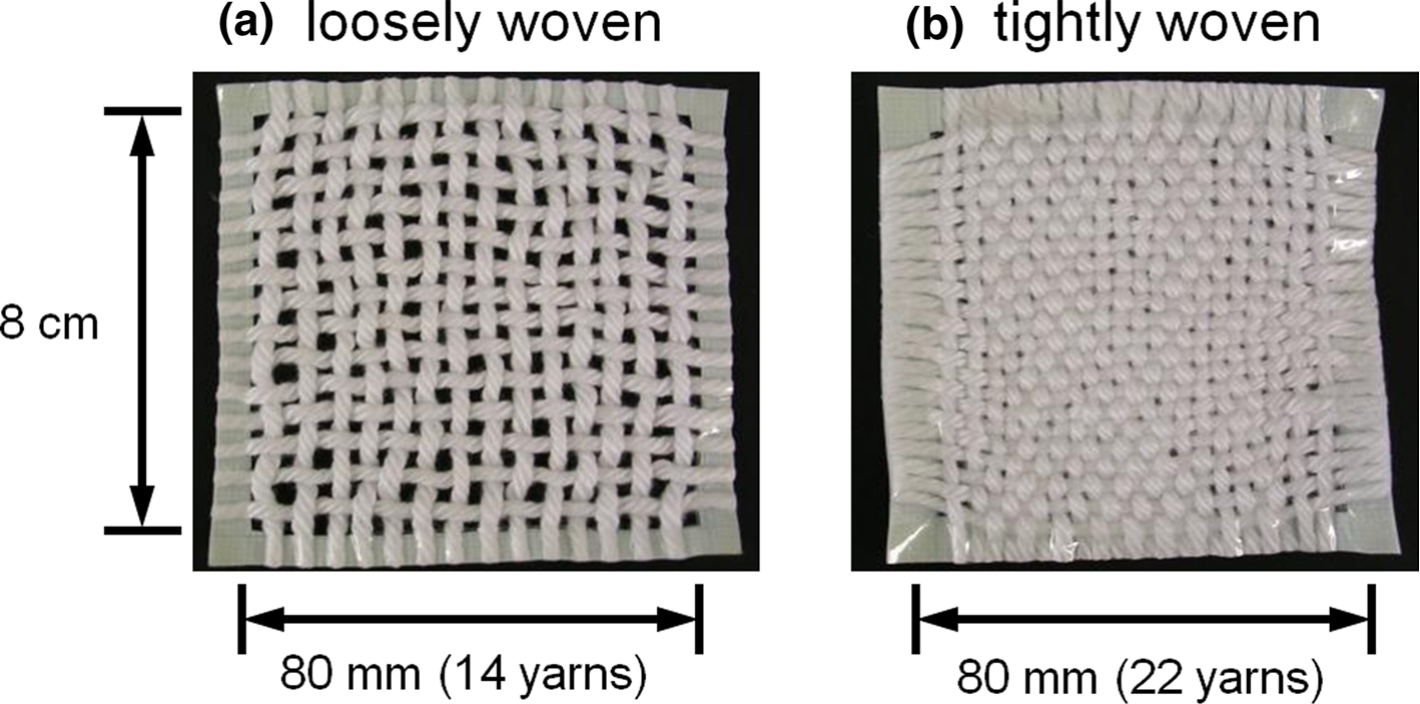
Model cloths

Two types of handmade cotton cloths, one loosely woven and the other tightly woven (Fig. 2), were prepared for use as models in the study. The cloths were cut into 8-cm^2^ squares.

#### Cotton Towels

Cotton towels (TW-220, Takei Corp., Japan) (Table 1) were also used as samples. Before the experiments, the samples were pre-washed in a fully automatic washing machine. Twenty-four cotton towels and 52.22 g of nonionic detergent (Emulgen108, Kao Corp., Japan) were placed in 47 L of water and washed in two steps as follows: (1) washing for 9 min using the detergent, rinsing twice with 47 L of water, and spin drying for 3 min (this step was carried out three times); (2) washing for 9 min with water only, rinsing twice with 47 L of water, and spin drying for 3 min (this step was carried out twice).

**Table 1:** Details of towel samples

Yarn fineness^a^			Fabric density^a^		Pile ratio^b^	Twist of Pile yarn (turns/m)^b^
Ground warp (tex)	Weft (tex)	Pile warp (tex)	Warp (ends/cm)	Weft (ends/cm)		
29.6	29.6	29.6	21.5	15.5	5.5	712

*JIS* Japanese industrial standards

^a^JIS1095

^b^JIS4105

### Softener Treatment

Distearyl dimethyl ammonium chloride (DODAC; Tokyo Kasei Corp., Japan) and Esteramide (EA; Kao Corp., Japan) were used without purification as the model softeners. The concentration of softener used for treatment of the yarns and cloths was chosen according to the testing conditions described in the following softener-treatment experiments.

#### Softener Treatment for Thick Cotton Yarn

Ion-exchanged water (400 mL) and a 0.5 % softener dispersion (0.8 mL) were placed in a 500-mL beaker and stirred for 1 min. The pre-washed thick cotton yarns (4 g) were immersed in this aqueous solution and stirred for 48 min (the bath ratio was set to 100). The treated wet yarns were spread on a sheet of polypropylene (PP; PC-8186, Sekisui Chemical Co.,Ltd., Japan) and allowed to dry naturally.

#### Softener Treatment for Model Cotton Cloths

Ion-exchanged water (400 mL) and a 0.5 % softener dispersion (0.8 mL) were placed in a 500-mL beaker and stirred for 1 min. Three pieces of the woven model cotton cloths were immersed in this aqueous solution and stirred for 5 min (the bath ratio was 100). The treated wet cloths were spread on the PP sheet and allowed to dry naturally.

#### Softener Treatment for Cotton Towels

Two pre-washed cotton towels (78 g each) were treated with an aqueous solution of the softener in a small washing machine (MiniMini Washer NA-35, Panasonic Corp., Japan). The bath ratio was set to 30, and 47 L of tap water (Wakayama city, Japan) was used at 25 °C. First, the softener [0–0.3 % on the weight of fabric (o.w.f.)] was dispersed in the water with sufficient stirring; then, two towels were immersed in the water and stirred for 5 min. Preliminary studies [1, 2] showed that the rate of softener adsorption on the fibers of these towels reached nearly 100 % within 5 min. Following treatment with the softener, the towels were spin-dried in a two-tank washing machine (PS-H35L, Hitachi Corp., Japan) for 3 min and manually fluffed five times. Samples from the pile area of the towels were used to measure the shear, friction, compression, and tensile properties.

### Measurement of Amount of Adsorbed Softener

#### Extraction of Softener Adsorbed on Yarns

Methanol (80 g, high-performance liquid chromatography [HPLC] grade; Wako Pure Chemical Industries, Ltd., Japan) and HCl (0.8 g, 35 % aqueous solution; Wako Pure Chemical Industries, Ltd., Japan) were poured into a 100-mL glass container. The thick cotton yarns (around 1.0 g) that had been pre-washed, treated with the softener, and dried were then added and subjected to ultrasonic agitation for 20 min. The resulting liquid was diluted with methanol, as required, until it reached 1/100th to 1/1000th of its original concentration. The amount of softener adsorbed on the yarns was measured three times by HPLC-mass spectrometry (HPLC–MS).

#### Equipment and Measurement Conditions

The amount of softener adsorbed on the cloths and yarns for this study was measured by an HPLC–MS (Prominence UFLC; Shimazu Seisakusho, Japan) system and an MS system (LCMS-2010; Shimazu Seisakusho, Japan) with electrospray ionization (ESI) in positive mode. The ionization conditions for electrospraying were optimized by the automatic calibration system. The analytical column, Unison UK-C18 HT (diameter 2 mm × length 50 mm, diameter of gel particles: 3 μm; Imtakt, Japan), was operated at 40 °C. A 10-mM aqueous acetic ammonium solution was used as mobile phase A, and a 10-mM acetic ammonium MeOH solution was used as mobile phase B. A gradient was set from 50 to 100 % B over 2 min and maintained for 3 min at a flow rate of 0.5 mL/min. For selected ion monitoring (SIM) measurements, the MS ionization mode was set as positive, and protonated molecular ions, [M+H]^+^, were used: mass to charge ratio, *m*/*z* = 550.7 for DODAC in the SIM mode.

### Observation of the Adsorption State of the Fabric Softener

**Fig. 3:**
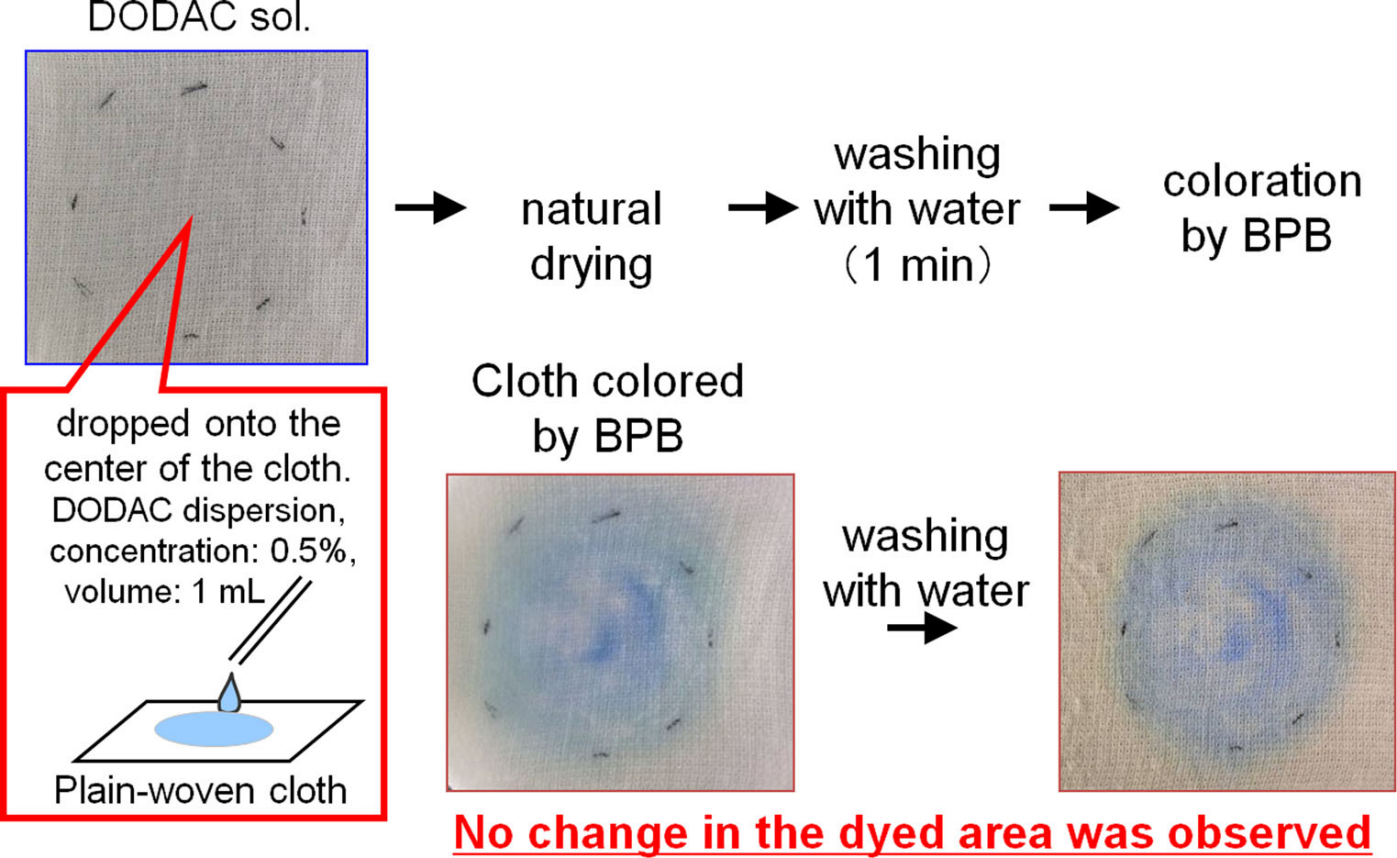
The coloration method with BPB

The model cotton cloths and thick cotton yarns that had been treated with the softener were completely immersed in an aqueous solution of bromophenol blue (BPB), a known indicator of cationic surfactants [10]. Color appeared on parts of the cloth where the cationic surfactant was adsorbed (Fig. 3). The aqueous BPB solution was prepared by mixing 75 mL of 0.2 N sodium acetate, 925 mL of 0.2 N acetic acid, and 20 mL of a 0.1 % ethanol solution of BPB (Wako Chemical, Japan).

### Evaluation of Color Intensity by the BPB Method

The test cloths treated with the aqueous BPB solution were cut to unravel the woven yarns (each piece was 8 cm long). The photographic images of the yarns were recorded by a microscope (SMZ-U, Nikon, Japan) under fixed illumination. The color of pixels composing the images was represented in the sRGB color space. The chromaticity parameters of red, green, and blue can be transformed to CIE XYZ tri-stimulus values [11]. We used the chromaticity coordinate *z* given in Eq. (1) as a suitable parameter for indicating the blueness of the pixels:

1 $$z = Z/\left( {X + Y + Z} \right)$$

where *X*, *Y*, and *Z* are the tri-stimulus values of each pixel. The *z* value of the standard illuminant D65 is 0.358, which corresponds to the *z* value of white. The distribution of *z* was calculated in eight squares (1.4 mm × 1.4 mm) for each yarn.

### Shear Property Measurements by the KES (Kawabata Evaluation System) Method

The shear properties of each sample were measured using the Kawabata evaluation system (KES). An automatic shear tester (KES-FB-1-AUTO-A; Katotech Corp., Japan) was employed, and samples were sheared under a load of 10 gf/cm^2^ at a shear rate of 0.417 mm/s. The hysteresis of shear force (2HG, measured in gf/cm) at a shear angle of 0.5° was obtained during the dynamic process at 25 °C and 50 % RH. As the value of 2HG became higher, the sample showed less resilience. The measurements were carried out six times.

### Frictional Property Measurements by the KES Method

An automatic frictional tester (KES-FB-4-AUTO-A; Katotech Corp., Japan) was employed to carry out frictional property measurements on each sample. During the measurement, 20 gf/cm of tension was added, and the contact device made of piano wire (the weight of the load was approximately 50 gf) was moved over a length of 2.5 cm at a speed of 1 mm/s on the pile. In doing so, the average mean coefficient of friction (MIU) was measured at 25 °C and 50 % RH. The measurements were carried out six times.

### Compression Property Measurements by the KES Method

An automatic compression tester (KES-FB-3-AUTO-A, Katotech Corp., Japan) was employed to measure the compression properties of each sample. The samples were compressed under a maximum force of 50 gf/cm^2^ at a compression speed of 0.02 mm/s. The work of compression (WC, gf/cm) was measured during the dynamic process at 25 °C and 50 % RH. As WC increased, it became easier to compress the sample. The measurements were carried out six times.

### Tensile Property Measurements by the KES Method

An automatic tensile tester (KES-FB-1-AUTO-A; Katotech Corp. Japan) was employed to carry out tensile property measurements on each sample. The samples were measured under the maximum load of 500 gf/cm^2^ at a tensile rate of 0.2 mm/s. The tensile energy (WT, gf/cm) was measured during the dynamic process at 25 °C and 50 % RH. As WT increased, it became easier to stretch the sample. The measurements were carried out six times.

### Evaluation of Yarn Pull-Out Force from a Plain-Woven Cloth

One piece of yarn from a 7 cm × 3 cm area of the plain-woven cloth was cut from the middle position, up to 2 cm from the bottom of the cloth, and pulled out from the cloth. The pull-out force was measured using Sebastian’s method [12] at a speed of 1 cm/min with a universal testing machine (TENSILON, A&D, Japan).

### Adsorption State of the Softener

Staining tests using a 4 % aqueous osmium tetroxide solution were carried out both for the softener on a glass plate and on the model yarns (100-fold concentration; 10 % o.w.f.). Effective staining of the yarn was visually confirmed. The softener-treated model yarn was steam-stained with a 4 % aqueous osmium tetroxide solution and embedded in a visible-light-curable resin (D-800, JEOL, Japan). A smooth cross section was then prepared using a microtome (Leica, Germany) for further analysis. Field-emission scanning electron microscopy (FE-SEM; JSM-7600F, JEOL, Japan) observations (10 kV), energy-dispersive X-ray spectrometry (EDS; INCA Energy, Oxford Instruments, UK) analysis (10 kV), and time-of-flight secondary ion mass spectrometry (TOF–SIMS; TOF–SIMS IV, IONTOF, Germany) analysis (25 kV) were performed. SEM observation using a backscattered electron detector showed good contrast corresponding to the element number. In terms of both sensitivity and resolution, the TOF–SIMS results gave the best overview of the distribution of softener molecules in the cross section.

### Evaluation of Softening Effect Using Model Cloths

The level of softness of the model cloths was evaluated via sensory tests performed by five expert panelists. The evaluation was carried out by compression of the cloth between the panelists’ palms.

### Evaluation of Softening Effect Using Towels

Softener EA (Esteramide, Kao Corp., Japan) was used for preparing reference towels to determine the level of softness of the tested towels. The concentrations of EA were set to six grades: 0, 0.025, 0.05, 0.075, 0.1, and 0.125 % o.w.f. (the softening levels corresponding to each grade were denoted on a scale of 1 to 6). The level of softness was evaluated by five panelists through comparison sensory tests using the reference towels.

## Results and Discussion

### BPB Coloring Method for Determining the Softener Adsorption Area

First, 1 mL of 0.5 wt % aqueous softener dispersion was released in drops onto the central part of a plain woven cloth (5 cm × 5 cm). The dispersion area was then marked using a permanent marker, and the cloth was allowed to dry naturally. The cloth was then washed in 3 L of ion-exchanged water, followed by immersion in the BPB coloring reagent. BPB is known to be a good indicator of cationic surfactants, and we were able to visually identify the area in which the softener was adsorbed. The resulting blue-stained areas indicated the presence of the softener. We found that the softener did not wash off or drift from its original position after the sample was cleaned with ion-exchanged water. Thus this coloring method is useful for identifying areas on a sample where the softener exists.

### Role of the Yarn Cross-Points in the Cloth in Relation to the Softening Effect

#### Postulate #1: The Softener at the Cross-Point of a Cloth Did Not Play an Influential Role in Overall Softening

**Fig. 4:**
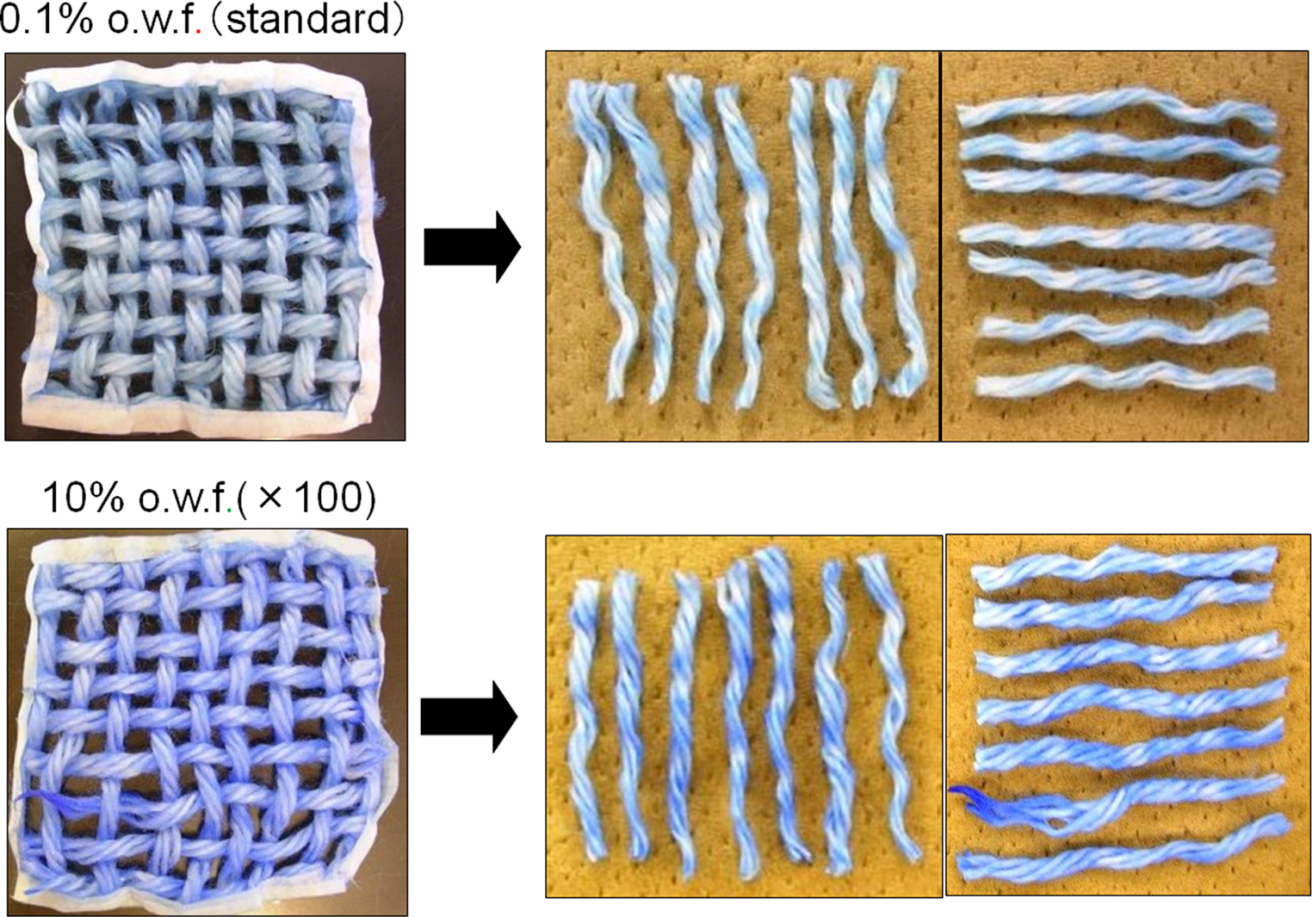
The appearance of the yarns after BPB coloration

When a model cotton cloth treated with a standard concentration (0.1 % o.w.f.) of softener was soaked in the aqueous solution of the BPB coloring reagent, the cloth was stained blue, as expected. When the cotton model cloth was cut and unraveled into individual strands of yarn, however, we recognized the uneven color in these strands, and the expected blue coloration at the cross-point surfaces was not observed. Almost the same unevenness was obtained when a 100-fold higher concentration (10 % o.w.f.) of the softener was used (Fig. 4).

**Fig. 5:**
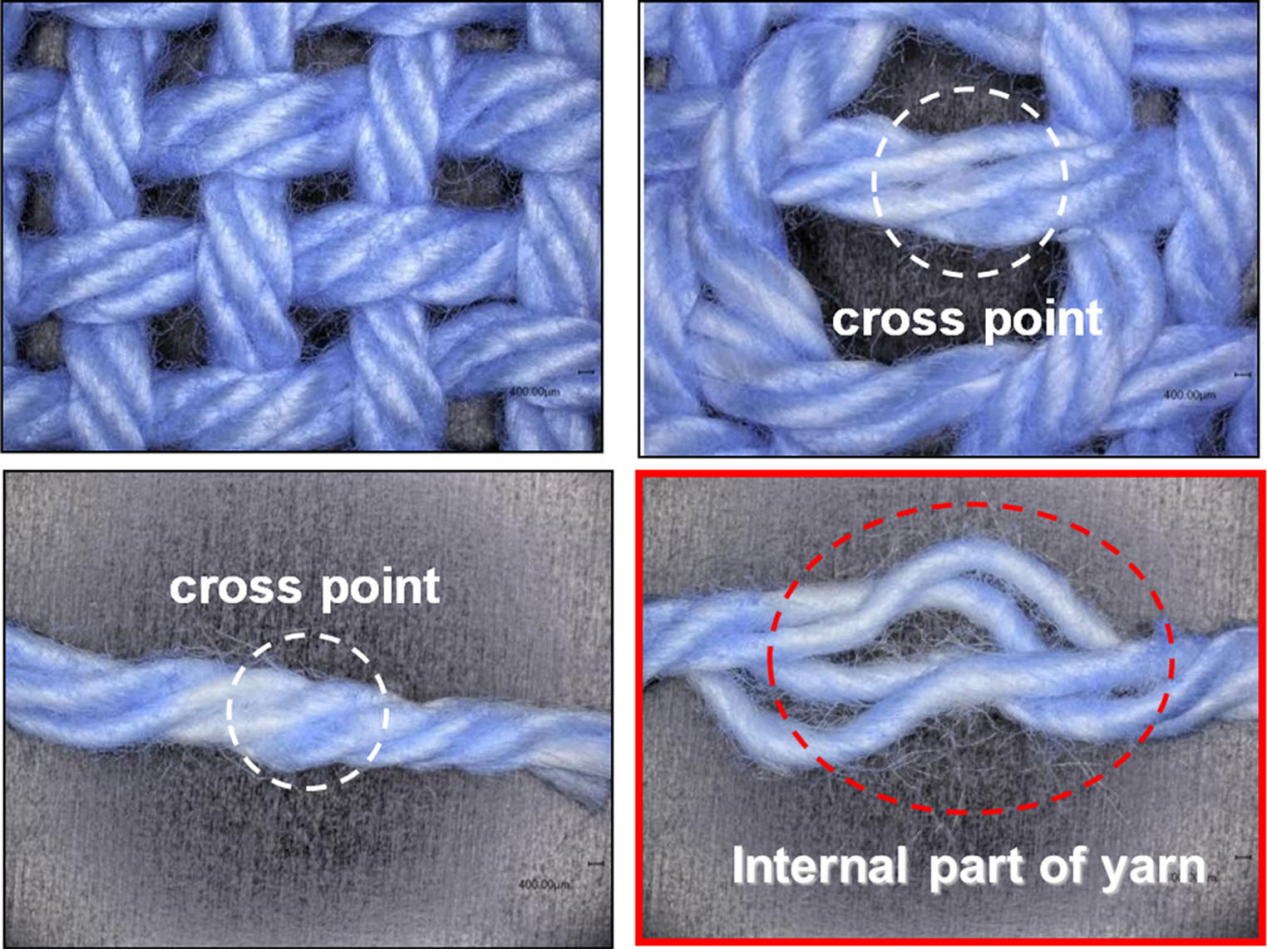
Adsorption states at the cross-point using a softener

These results lead us to conclude that the softener was present in low amounts at the yarn cross-points and was preferentially adsorbed on the exposed surface. In addition, the coloration state was observed by untwisting a colored thick yarn obtained from the cut model cloth. The results are shown in Fig. 5. The color intensity of the inner part of a strand of yarn was weaker than that of the outer part. Based on these experimental results, we conclude that the softener had a tendency to be adsorbed primarily on the exposed parts of the cloth and the yarns.

**Fig. 6:**
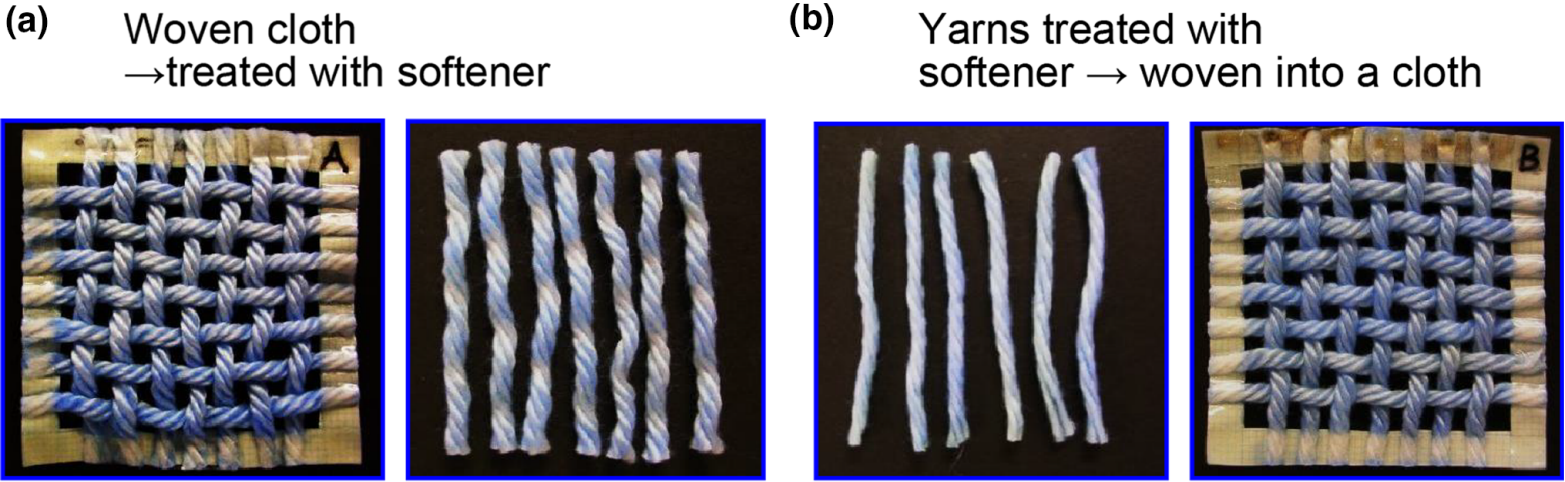
Comparative evaluations of softening effect of samples (a) and (b)

Next, we compared the softening effect using samples with different adsorption patterns (Fig. 6). In sample (a), the concentration of softener at the cross-points was low; the cloth was woven, treated with the softener, and dyed with BPB. In sample (b), the concentration of softener at the cross-points was high; the yarns were first treated with the softener, then woven into a piece of cloth, and dyed with BPB.

To accurately compare the two samples, the amount of adsorbed softener must be set to the same level. When sample (a) was prepared, we stirred for 5 min to allow the softener to be adsorbed on the cotton surface. This is the same condition as that used to prepare the model cotton towels. When the HPLC–MS was used, the resultant amount of adsorbed softener was 762 mg/kg (corresponding to an adsorption rate of 76.2 %). However, when sample (b) was prepared, the amount of adsorbed softener was as low as 369 mg/kg (the adsorption rate was 36.9 %), despite stirring the sample for the same amount of time. To overcome this imbalance, the yarn treatment time was increased to 48 min, at which point the amount of softener adsorbed by sample (a) was 800 mg/kg. The significant difference between the adsorption rates of these two samples might have been caused by the difference in the frequency of collisions between vesicles and fibers. This collision frequency was lower in the yarns because they drifted more easily, synchronizing with the flow of the aqueous softener solution.

Based on the above results, we can interpret the mechanism of softener vesicle adsorption on fibers as “adhesion by collision,” which supports the findings of Minegishi [6] and Sakai et al. [9]. In this mechanism, the softener has a tendency to be adsorbed on the exposed parts of the fibers or cloth sample, because the adsorption is controlled mainly by the collision of the softener vesicles with the fibers.

We then tried to compare the softening effect by performing sensory tests (*n* = 3) using samples (a) and (b) (Fig. 6). Before the tests, we confirmed that there was no difference between the two samples when no softener was used.

**Fig. 7:**
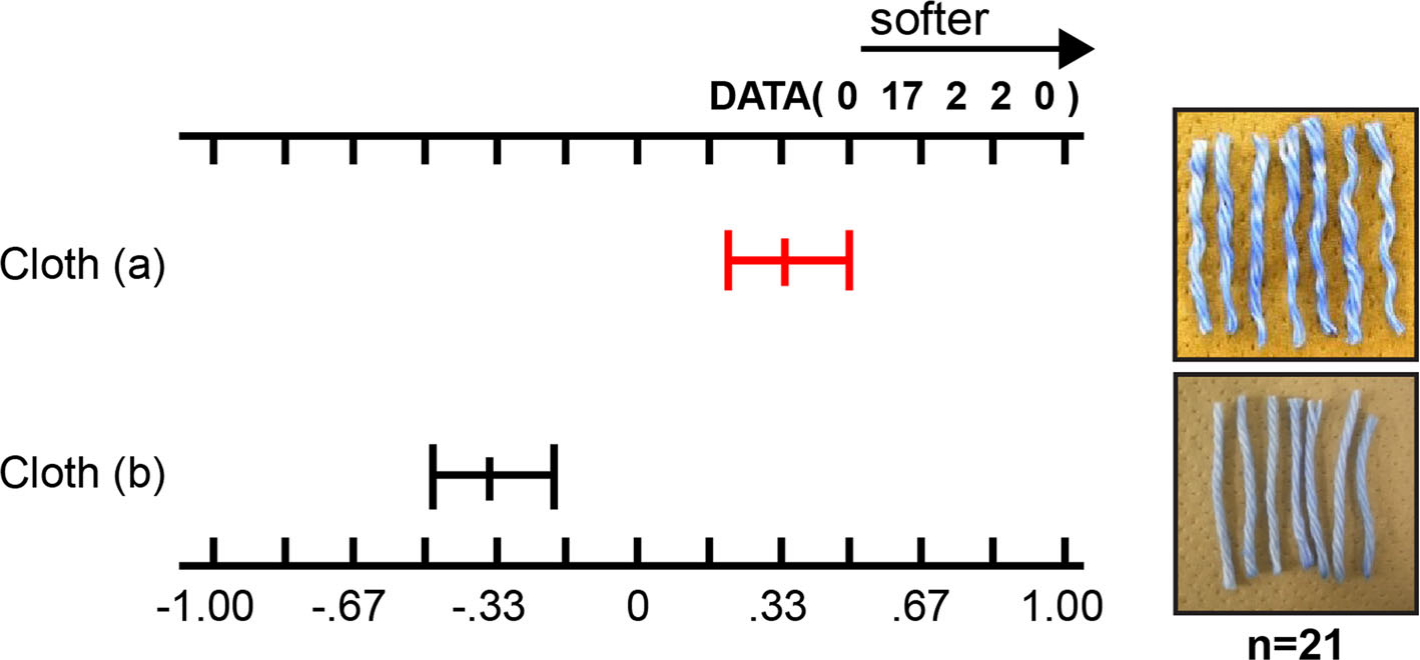
Softening sensory test results comparing woven cloth samples (a) and (b)

When we compared the softening effects of samples (a) and (b) after using the softener, sample (a) unexpectedly showed higher softening and had a more bouncy feel (Fig. 7) than sample (b).

As mentioned before, sample (a) showed uneven adsorption of the softener. In other words, the blue coloration indicating the existence of softener molecules, i.e., cationic agents, was less prevalent at the cross-points in the yarns of sample (a). The fact that sample (a) showed higher softening clearly demonstrates that the adsorption of a softener at the cross-points in a cloth has no significant impact; however, adsorption on the exposed part of the cloth is important for effective softening. The reproducibility of this phenomenon was confirmed using model cotton cloths of different densities (loosely woven and tightly woven samples) (Fig. 2).

#### Postulate #2: The Softener at the Cross-Point of a Cloth Played an Influential Role in Overall Softening

The KES method for evaluating the shearing, surface, compressional, and tensile properties was applied to towels prepared by treatment with the softener at different concentrations (0–0.2 % o.w.f.).

**Fig. 8:**
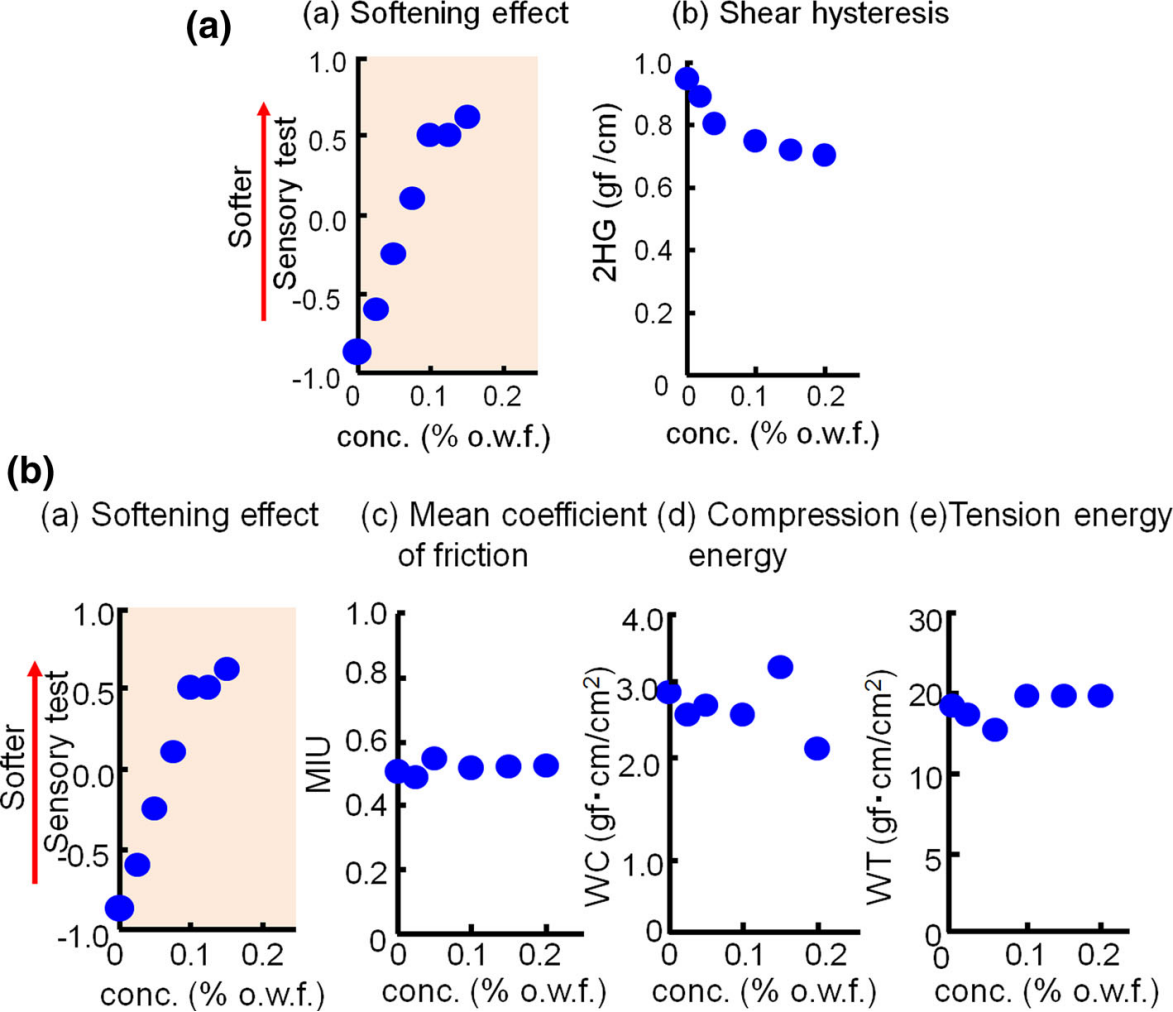
**a** Sensory test of softening and the shear hysteresis values obtained by KES methods vs. softener concentrations. **b** Sensory tests and other various types of values obtained by KES methods

**Fig. 9:**
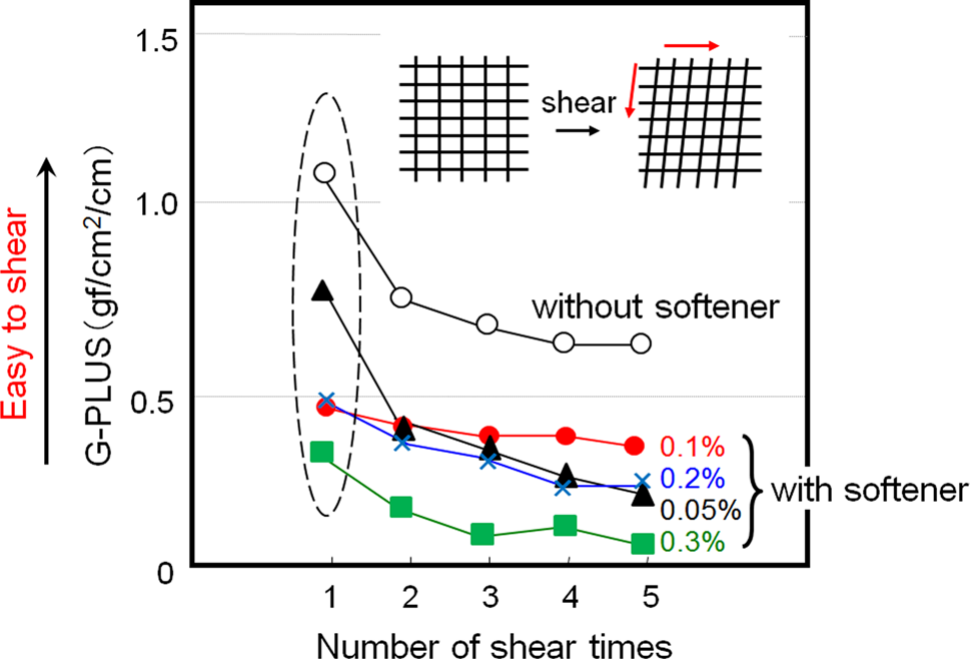
The results of shear force measurement using a softening agent at different concentrations

As shown in Figs. 8a, b and 9, only the shearing hysteresis was correlated with the softening effect. The shear hysteresis decreased with increasing softener concentration and softening effect. Surprisingly, we could not find any correlation between the softener concentration and the frictional force [(a) and (c) in Fig. 8a], which has been widely accepted as an important factor in cloth softening. On the other hand, the yarn cross-points in a cloth were considered to play some important role, because the shearing property had been interpreted as the ease of movement at the cross-points between the yarns. Here, in this Postulate #2, we faced with a contradiction.

**Fig. 10:**
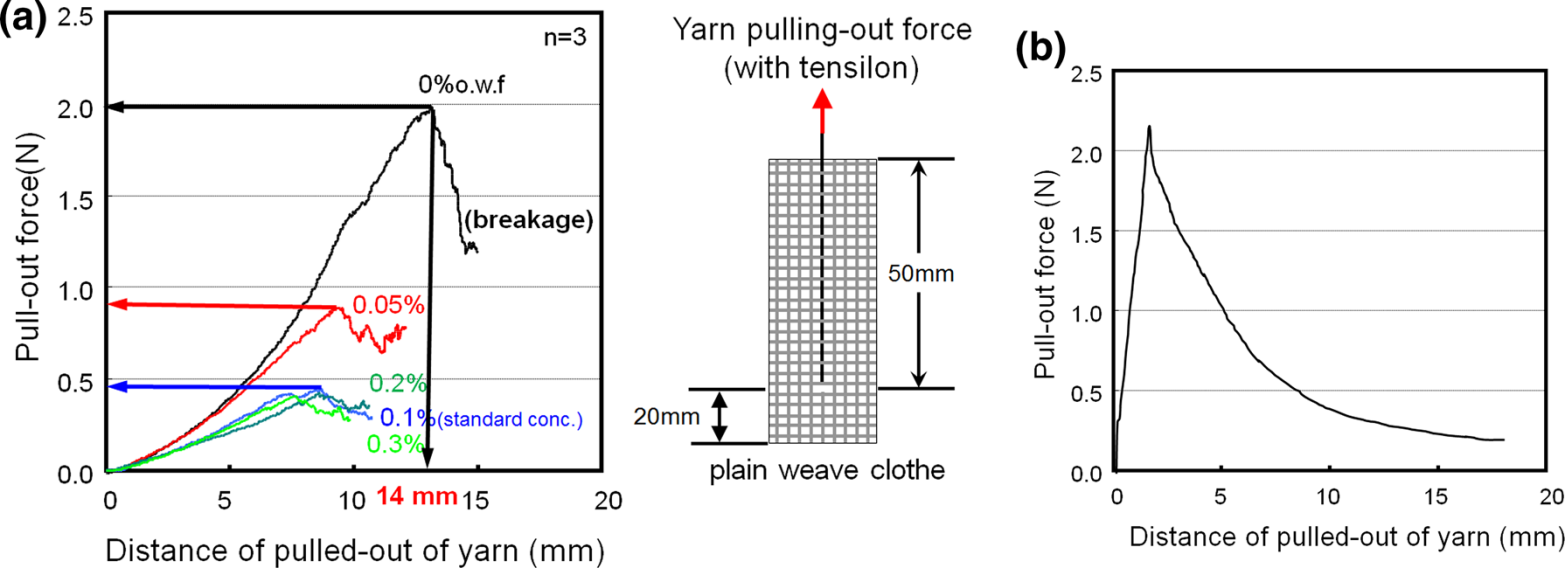
**a** The result of pull-out force corresponding to the distance of pull-out of yarn. **b** Pull-out force corresponding to the distance of pull-out of yarn

Then, the pull-out force measurement results were considered in order to evaluate the importance of the yarn cross-points in relation to the softening effect. With this method, we determined the maximum static frictional force needed to pull out one strand of yarn from the cloth. The pull-out force decreased with increasing concentration of the softener (Fig. 10a). This result also supports the theory that the presence of the softener at the cross-points has an important role in softening.

#### Interpretation of the Role of Cross-Points

The two results described under Postulates #1 and #2 lead to contradictory conclusions, i.e., “cross-points are not influential” versus “cross-points are influential” in the softening of a piece of cloth. Therefore, we examined the pull-out force measurement process more carefully, and an interesting fact was revealed. The point corresponding to the maximum pull-out force in the plain woven cloth sample was not located at the position immediately after the starting point of the pull-out process, but 14 mm away from the starting position, as shown in Fig. 10a (*left panel*), when the actual sample size was only 50 mm (Fig. 10a, *right panel*; the total size of the sample was 70 mm). This large shift, i.e., 14 mm, is quite aberrant if we compare it with pull-out measurement results commonly observed in other studies. When softeners of different concentrations were used (Fig. 10a), the same type of unusual shift was observed.

If we consider only the effect of static and kinetic friction between yarns or fibers because the number of cross-points in a cloth sample is the greatest at the very early stage of the pull-out action, the frictional force must have the greatest influence in this early stage. However, it should decrease gradually in the latter stages, such that the force curve should be similar to that shown in Fig. 10b, in which a peak appears in the very early stage. What we found in our experiment (Fig. 10a, *left panel*), however, was quite different.

**Fig. 11:**
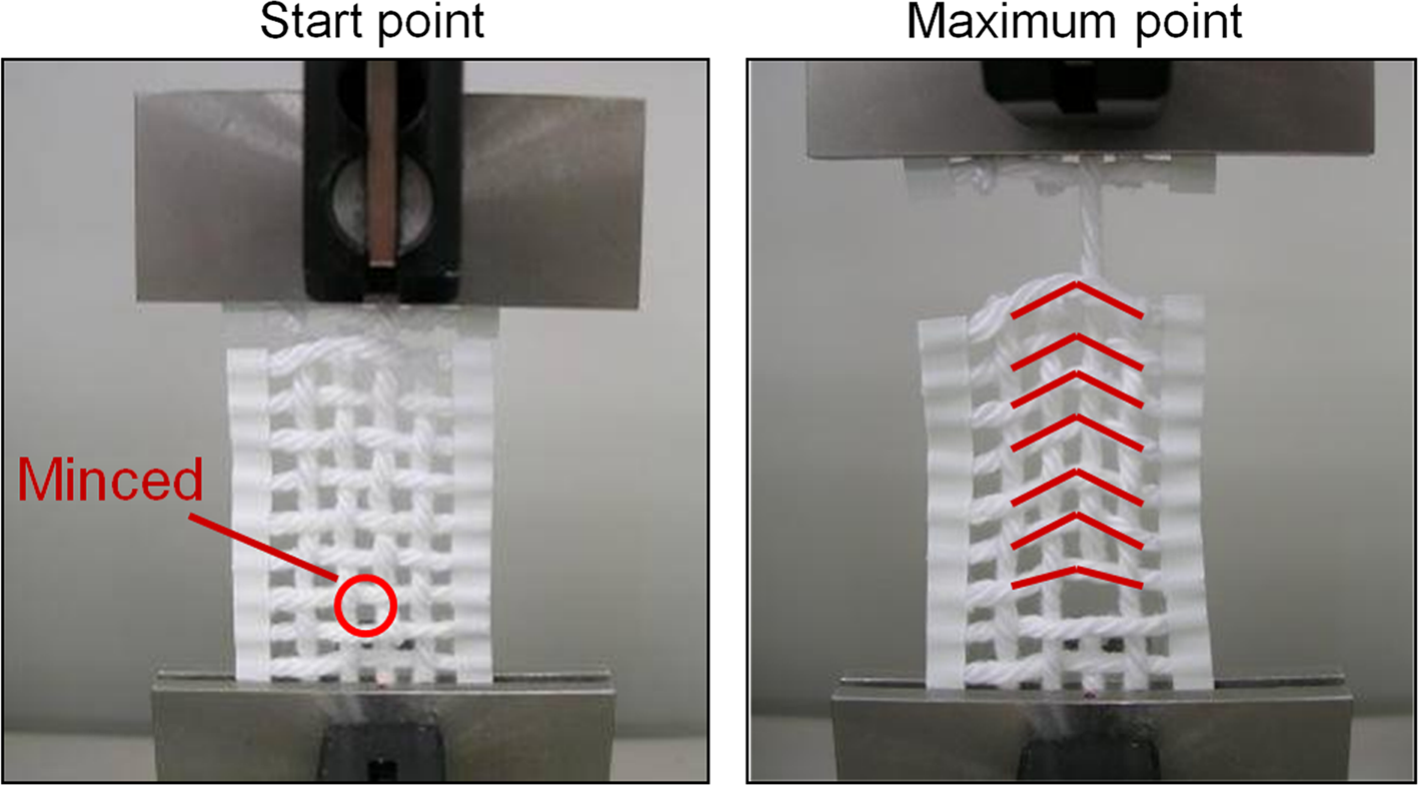
Photograph of pull-out test using Sebastian’s method

We then tried to visually observe the state of this pull-out measurement using a non-treated “model cloth” with thicker yarns (Fig. 11). The pulled-out vertical yarn appeared to adhere to the horizontal yarns, and deformation of this model cloth was obvious. Thus, the pull-out maximum force values obtained with this method (Fig. 10a) did not reflect the MIU obtained using the KES-type frictional forces. Therefore, this phenomenon is not related to lubrication, but it is related to “adhesion between yarns.” In the report by Sebastian et al. [12], the authors also mentioned that this phenomenon was possible to be caused by some adhesive force. This phenomenon can thus be explained on the basis of static friction or adhesion rather than sliding between the yarns.

To clarify the reason for the two contradictory theories about the role of cross-points, a hypothesis was developed and examined, as described below.

##### Hypothesis

**Fig. 12:**
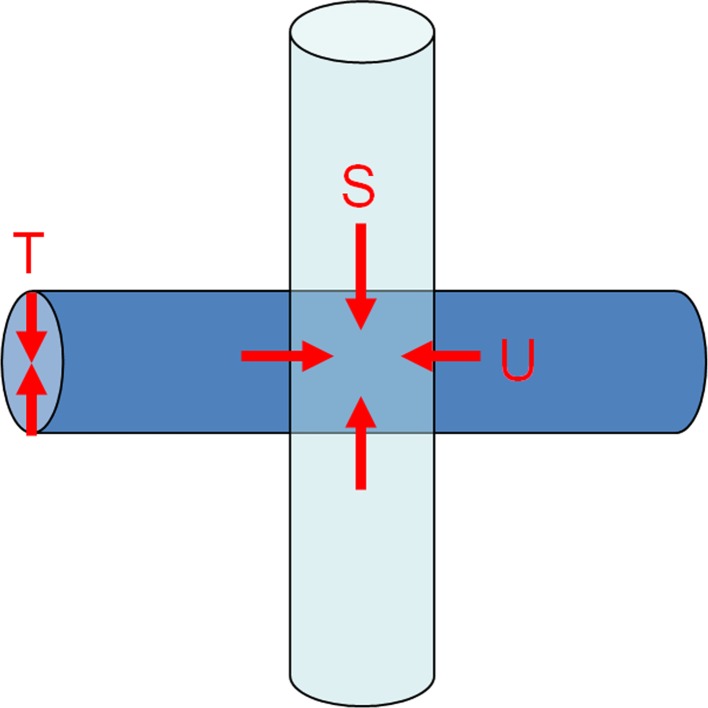
Image of gradual penetration of softener from *S*, *U* and *T* directions

The softener, which has vesicles considerably larger than the micelles, adheres to and is immobilized at the fiber surface, preferentially around the exposed part of the yarn, because of collisions with the fibers. When the concentration of the softener increases, the probability of diffusion of the softener into the inner area of the cross-point gradually increases (Fig. 12, along the *S* and *U* directions). Thus we can see the correlation between the softening effect and physical textile properties such as shear force and yarn pull-out force.

When no softener is used, full adhesion occurs by hydrogen bonding between the yarns or fibers [1, 2]. In the pull-out force measurement process, when one yarn is selected and pulled upward, deformation of the cloth structure occurs because this yarn is adhered to the horizontal yarns in the cloth sample until the hydrogen bonds begin to break. As a result, the peak of the pull-out force curve shifts to the right, as seen in the left panel of Fig. 10a. When a softener is used, the pull-out force decreases, but the shape of the force curve remains the same, and the shift of the peak to the right still occurs because some adhesion always remains.

When examining the shear, it is important to remember that hydrogen bonding and its breakage near the cross-points of the yarns is controlled by the presence of softener in the same manner as that shown in the pull-out force measurement results. However, we found that the softness was unrelated to the cross-points, as discussed under Postulate #1 and shown in Figs. 6 and 7. Thus the shear hysteresis or pull-out force is considered to be “indirectly” correlated with the softness, owing to the interposition of the hydrogen bonding network between the fibers or yarns.

In order to verify the above hypothesis, we tried to quantitatively evaluate the change in the degree of coloration in a strand of yarn from a model cotton cloth. The degree of permeation of the softener from the *S* and *U* directions was measured, as shown in Fig. 12.

**Fig. 13:**
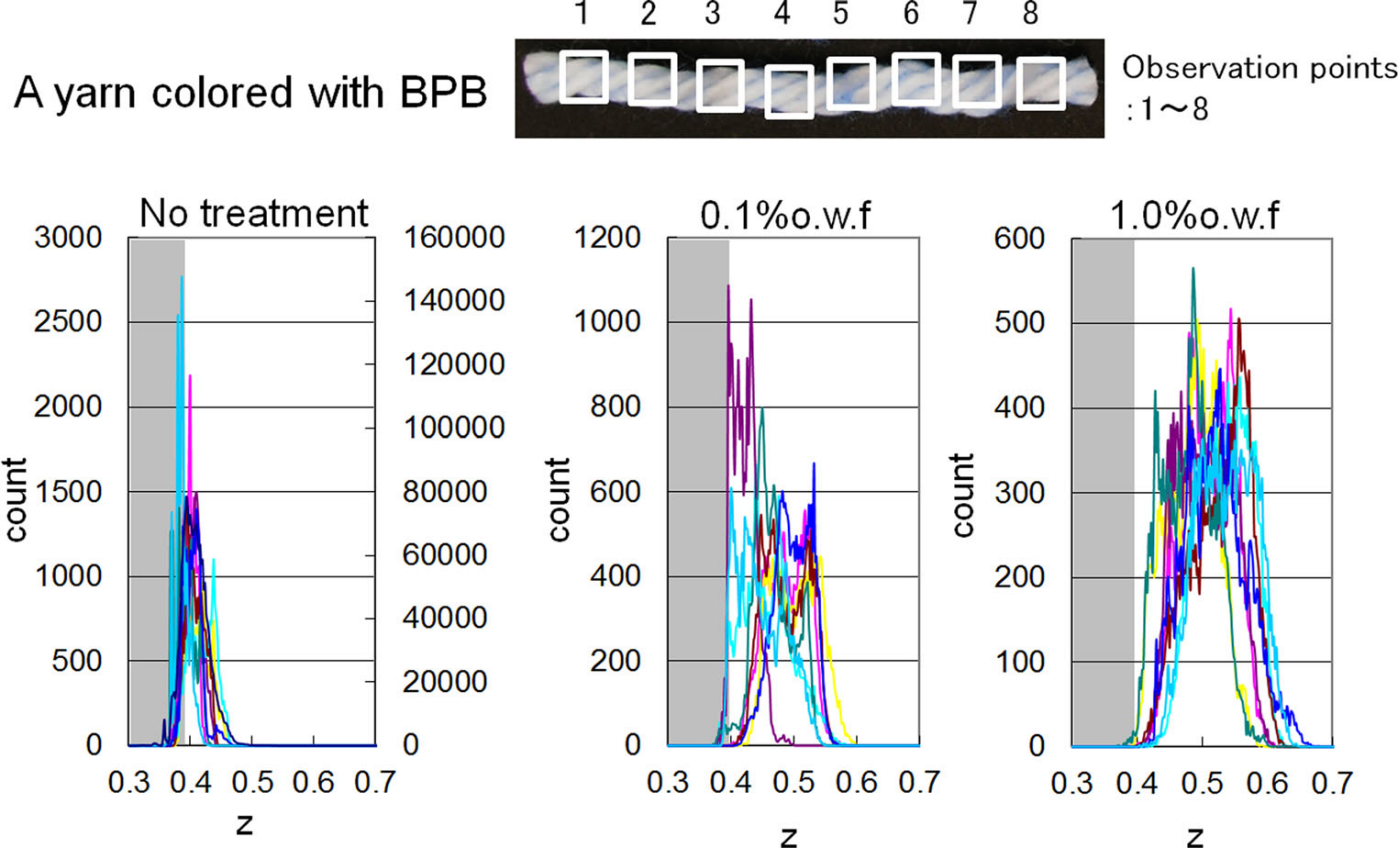
Results of intensity of chromaticity coordinate *z* obtained by IEC method without and with softener treatment (0, 0.1, 1.0 % o.w.f.)

In Fig. 13, the horizontal axis shows the parameter *z*, i.e., the chromaticity coordinate, which indicates the strength of blue coloration that correlates with the amount of adsorbed softener. When the coloration level is white, *z* equals 0.358 in this experiment. If the value of *z* increases, this means the concentrations of existing BPB and softener were increasing. The vertical axis shows the distribution of *z*, i.e., the number of pixels. As discussed earlier, when the softener concentration was 0.1 % o.w.f., the coloration at the cross-point was not significant, and the cross-points remained white. However, the size of the white-colored area decreased and the size of the blue-colored area increased in the model cloth when the softener concentration used was 10 times higher (1.0 % o.w.f.) than the standard level (0.1 % o.w.f.). These results show that the amount of softener gradually increased at the cross-points because of permeation and adhesion of the softener with increasing concentration. Thus, there is a correlation between the pull-out force and the softening effect with increasing concentration of the softener because of the gradual permeation of the softener in the *S* and *U* directions (Fig. 12), which decreased the hydrogen-bonding network—the origin of the adhesive force—between the fibers or yarns. The results obtained from the measurements of the shear force can also be interpreted as the breakage of this hydrogen bonding network because of the applied twisting force between yarns at the cross-points on the cloth.

### Observation of the Cross Section of a Model Strand of Yarn

We successfully stained a cross section of a strand of yarn treated with the softener with osmium tetroxide, and the yarn was partially blackened. Because the blackening was very weak for the untreated yarn, staining was found to be selective for the softener. Using osmium tetroxide staining, detailed SEM–EDS analysis of a single fiber was possible, but overall analysis at the yarn level with suitable sensitivity was difficult.

**Fig. 14:**
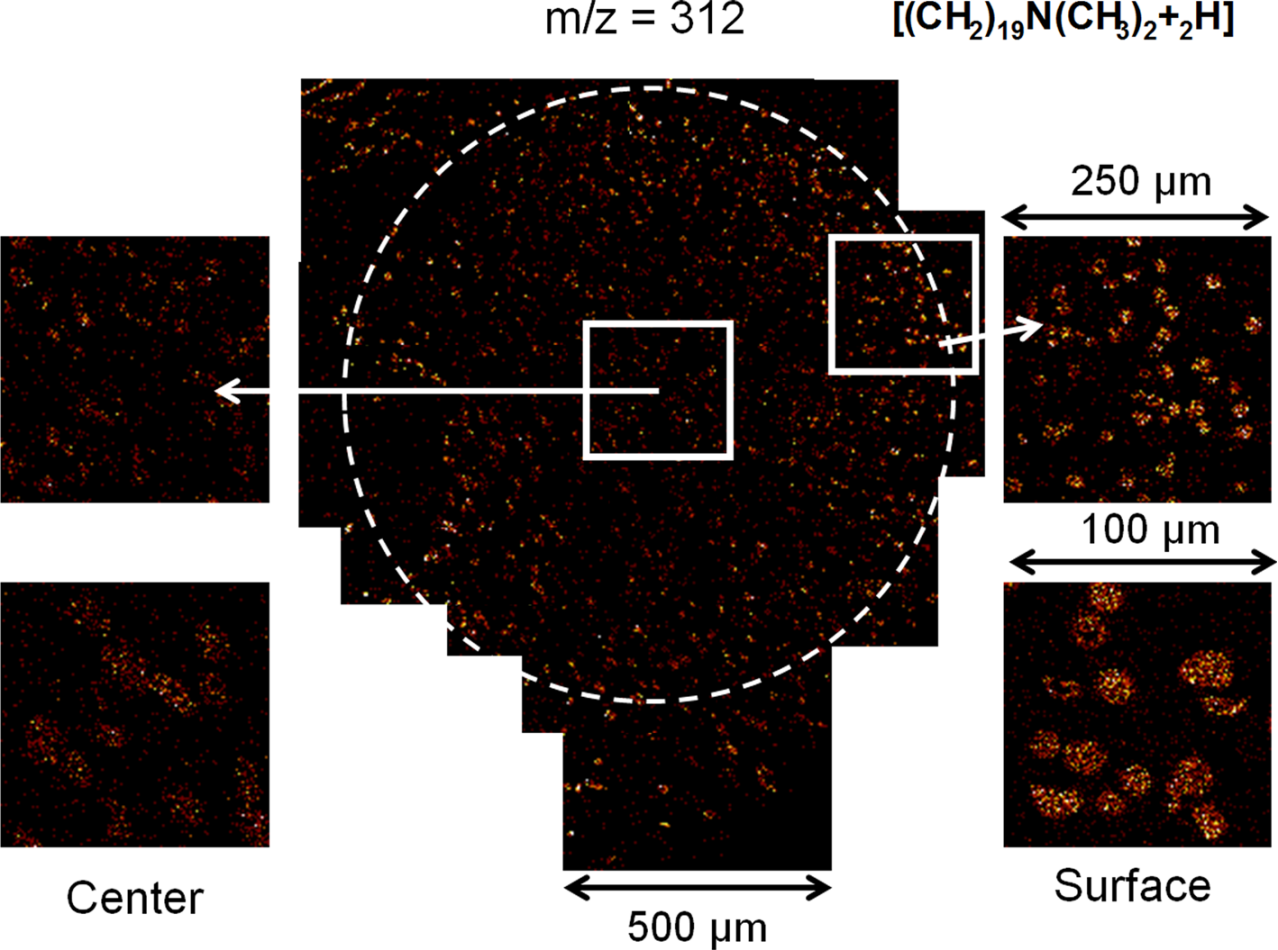
TOF-SIMS observation of the area where softener is present

Therefore, TOF–SIMS analysis, which has a higher sensitivity, was performed to detect a fragment of a softener molecule (*m*/*z* = 312), and the results are shown in Fig. 14. The concentration of the softener was higher near the surface of the yarn and lower toward the center of the yarn. This result is quite compatible with the findings indicating that the softener was mainly adsorbed over the exposed part of the yarn or cloth.

### Amount of Adsorbed Softener in the Inner and Outer Parts of a Thick Strand of Yarn

**Fig. 15:**
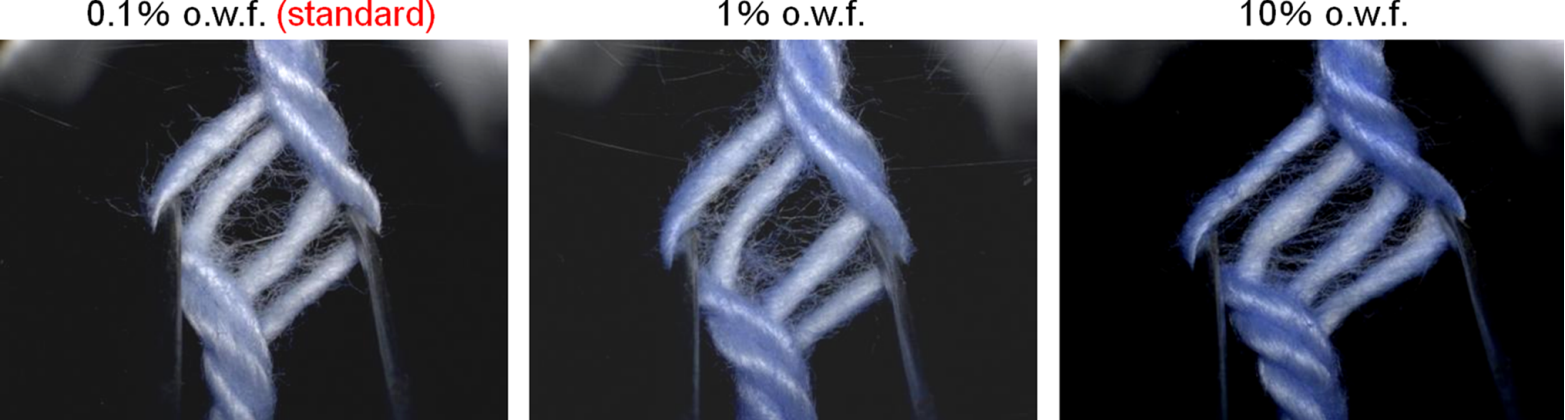
Distribution of softening agent inside the yarns

In order to further ascertain the presence of the abovementioned adsorption concentration gradation (Fig. 12, *T* direction), the difference in the strength of coloring between the outer and inner parts of a thick strand of yarn was studied simply by untwisting it: the coloration of the inner part of the yarns was weaker than that of the outer part in every case (Fig. 15) when the concentration of softener was increased from the standard level (0.1 % o.w.f.) to the 100-fold level.

**Fig. 16:**
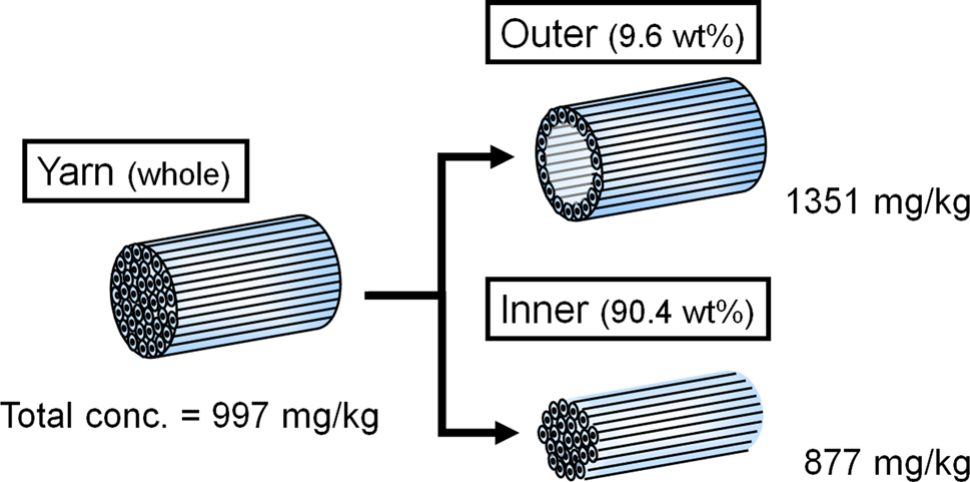
Gradational adsorption of the softening agent in a yarn

Another simple method was employed to show the presence of a concentration gradation in the *T* direction (Fig. 12). In total, 500 mg of yarn was pulled from the pile of a towel treated with the softener (at the standard concentration of 0.1 % o.w.f.). The outer part of the yarn was trimmed using sandpaper (around 10 wt% of the weight of the yarn was separated), and the amount of adsorbed softener was compared to that of the inner part by the method used to extract the adsorbed softener to determine its quantity. The total amount of adsorbed softener was 997 mg/kg. In the fractionated outer part (10 wt% of the total weight), the amount of adsorbed softener was 1351 mg/kg, and 877 mg/kg was found in the inner 90 % of the yarn. In other words, there was certainly less adsorbed softener in the inner part than in the outer part (Fig. 16).

Given such results, the softener was surely considered to be more concentrated on the surface or outer part of yarns than in the inner part. Moreover, the above-mentioned “gradation” actually existed.

### Relationship Between the Softener Concentration and Softening Effect

**Fig. 17:**
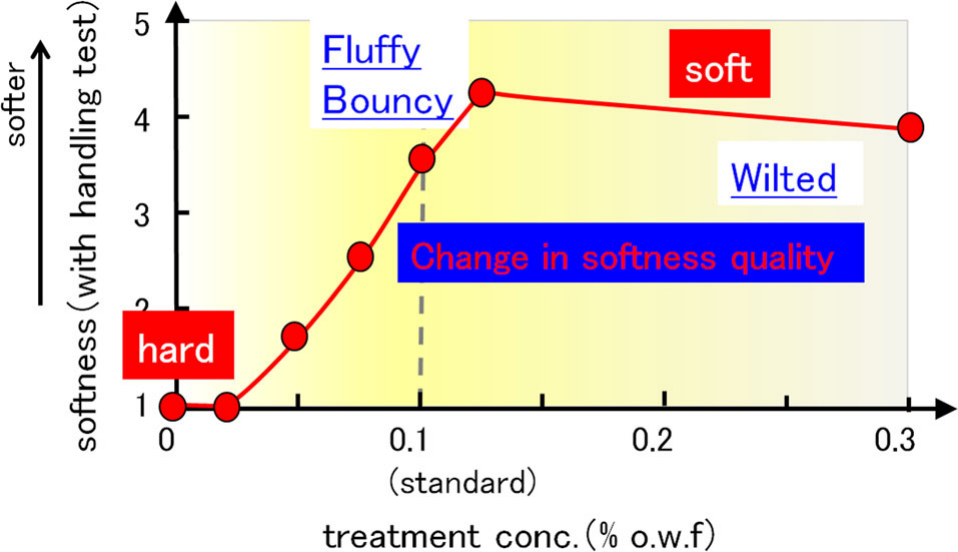
Relationship between treatment concentration and softening effect as determined by sensory tests

Figure 17 shows the relationship between the softener concentration and softening effect. In general, the softness increased from levels 1 through 4 when the concentration of the softener was increased to an approximately standard concentration (0.1 % o.w.f.) (see softening effect on towels by model softener EA). From this point until the concentration was increased to the three-fold level (0.3 % o.w.f.), there was no difference in the softness sensory evaluation scores (around level 4). However, when the “quality” of softness was compared between the standard-level and the excessive-level (three-fold level) concentrations in the same sensory tests, the difference was clear. The panelists found that the cloth was fluffy and bouncy when the standard concentration of softener was used, but they also reported the cloth as wilted when an excess amount of softener was used.

### Mechanism of the Softening Effect by Softeners

Based on the information hitherto described, a new mechanistic concept of the softening effect is proposed by considering that the cloth or yarns, or both, are an assembled architecture composed of fibers. As mentioned in Postulate #1, the amount of adsorbed softener was lower at the cross-point and in the inner part of the yarn. The softener was thus considered to have a tendency to be adsorbed on the exposed part of the fiber assembly.

This result shows that the softening effect of a softener is not caused mainly by lubrication at the cross-points of a cloth or between yarns. When we focused on one strand of yarn as an assembly consisting of single fibers, there was a difference between the amount of adsorbed softener in the inner and outer parts, i.e., gradation was observed.

**Fig. 18:**
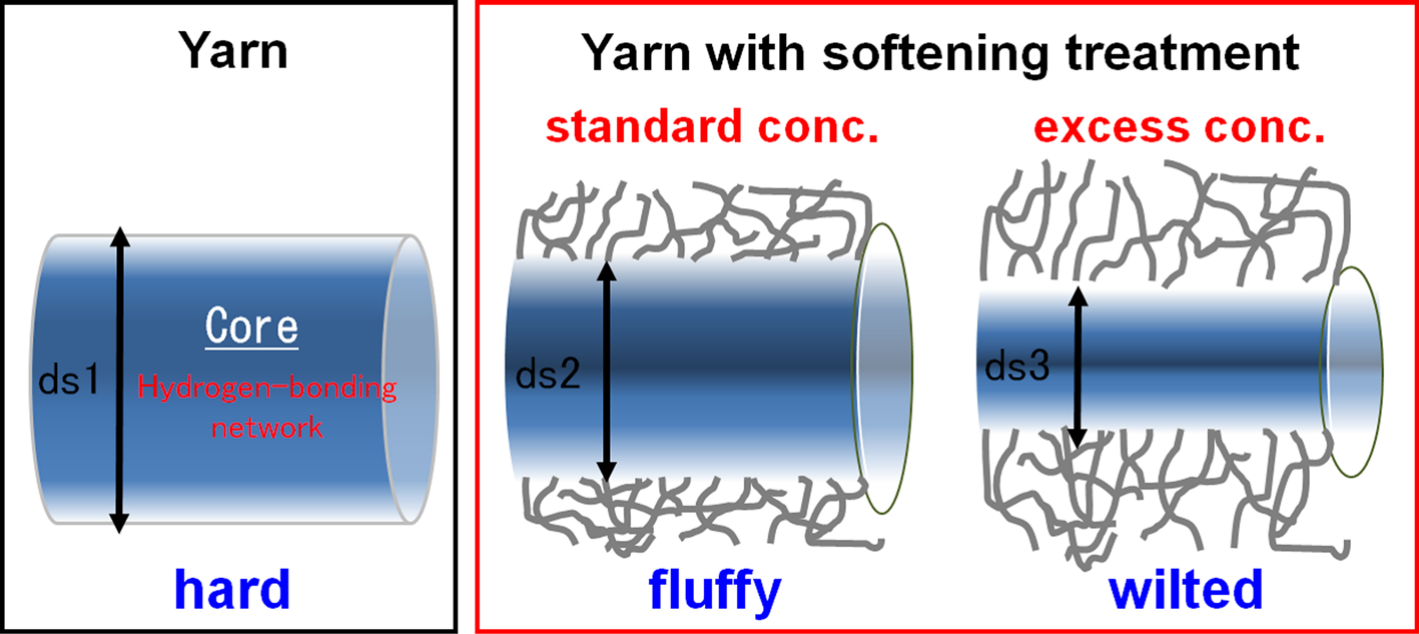
Image showing softening when different concentrations of softeners are used

Based on the above results, the main softening mechanism by the softener can be understood as shown in Fig. 18. First, a hydrogen-bonding network with bound water between fibers forms after natural drying, resulting in a certain degree of hardness. When the standard level of softener concentration is used for the cotton fibers, a high amount of adsorbed softener is present in the outer part, so that the formation of the hydrogen-bonding network is inhibited mainly around the outer or exposed part of a strand of yarn, resulting in a softening effect. Thus this area plays an important role in realizing the resultant “soft and fluffy structure.” On the other hand, the softener concentration is lower in the inner part of a strand, which plays an important role in imparting proper hardness to the yarn.

A good balance of both of these states is important for realizing the proper bouncy feel. Using a rough model in which a strand of yarn is imagined as a cylinder, as shown in Fig. 18, all parts of the yarn are hardened by the cross-linking of the hydrogen-bonding network when no softener is used. When a proper amount of softener is used, a good balance of a soft and fluffy outside and a hard inner side are realized, resulting in the bouncy feel. Furthermore, when an excess amount of softener is used, it diffuses into the inner part of the yarn, reducing the diameter of the cylinder. Because the rigidity of the cylinder is proportional to the fourth power of the diameter, the slight difference in the diameter causes a significant difference in the rigidity. Moreover, when the yarn is treated with an excess amount of softener, the core diameter decreases, the bouncy feel is not obtained, and the yarn wilts.

Softener adhesion to the cotton fiber assembly in the cloth and yarn was observed using the coloration method. The softener concentration was found to be very low at the cross-points of the cloth and high in the exposed part of the fiber assemblies. These results coincide with the mechanisms reported by Minegishi [6] and Sakai et al. [9], according to which the softener is considered to be adsorbed on the fiber surface because of collisions and subsequent adhesion of the vesicles.

When we investigated the softness of the model cloth in which the softener was present mainly on the exposed part and rarely at the cross-points (unevenly treated cloth), this cloth was found to be softer than the cloth in which softener was evenly adsorbed. The cloth containing less softener at the cross-points was apparently softer, contrary to the “lubrication-based” expectation. In other words, these results support the idea that increased sliding at the cross-points using softener is not necessary, even if the lubrication theory is considered to hold true based on the results of shear force and yarn pull-out force measurements. Furthermore, when we investigated the amount of softener adsorbed in the inner and outer parts of the yarn, the concentration in the outer part was higher, and there was a concentration gradient from the outer to the inner side. Thus, the softener was easily adsorbed on the exposed part of the yarn.

Based on these results and our preliminary studies [1, 2], we propose the following new softening mechanism for fabric softeners. First, the formation of the hydrogen-bonding network by bound water between cotton fibers is effectively inhibited by the preferential adsorption of softener at the outer part of yarns or cloths. In contrast, softener adsorption in the inner part of a strand of yarn is lower and the structure is harder because of the hydrogen bonding. Thus, the appropriate hardness is maintained in the inner part. Because of the good balance of these two phenomena, the favorable bouncy feel for the cloth or yarns is realized. When an excess amount of softener is used, however, the softener that is originally adhered to the exposed surface of the strand gradually penetrates the inner part of the strand, decreasing the hydrogen bonding, which in turn results in a reduction in the hardness and bouncy feel. This penetration inside the yarn is considered to be the cause of the wilted feel of a cloth with excess softener.

## References

[1] Igarashi T, Nakamura K, Okamoto Y, Asami N (2011) The 63rd divisional meeting on colloid and surface chemistry, Chemical Society of Japan, 7–9 September

[2] Igarashi T, Nakamura K, Okamoto Y, Morita N (2015). Elucidation of softening mechanism in fabric softener. J Surfactants Deterg.

[3] Evans WP (1969). Cationic fabric softeners. Chem Ind.

[4] Miyasaka H (2005). Recent trends in development of domestic fabric softeners. Oleo Sci..

[5] Crutzen AM (1995). Study on ditallow dimethyl ammonium chloride interaction with cellulose. J Am Oil Chem Soc.

[6] Minegishi Y, Arai Y (1977). Recent studies on cationic surfactants as domestic fabric softener. J Oil Chem Soc Jpn.

[7] Okumura O, Ohbu K, Yokoi K, Yamada K, Saika D (1983). A study on the adsorption of dialkyldimethyl ammonium chloride. J Am Oil Chem Soc.

[8] Nakamura K, Fujisawa K, Kunieda H (1997). Adsorption of double-chain cationic surfactant on hydrophilic surface. J Oil Chem Soc Jpn.

[9] Sakai T, Matsumoto K, Inoue S, Sonoi A, Ando T, Uchihashi T, Ando T (2012). IACIS.

[10] Takahashi E, Nanba Y, Koike M, Kobayashi M (2000) Handbook of surfactants, Kougaku tosho Corporation (Tokyo, Japan), pp 370

[11] IEC61966-2-1:1999, Multimedia systems and equipment—color measurement and management—Part2-1:Color management—default RGB color space

[12] Sebastian SARD, Bailey AI, Briscos BJ, Tabor D (1986). Effect of a softening agent on yarn pull-out force of a plain weave fabric. Text Res J.

